# Prevalence of Disordered Eating Risk Attitudes in Youth Elite Male and Female Football Players

**DOI:** 10.3390/jcm13206178

**Published:** 2024-10-17

**Authors:** Fernanda Vásquez-Díaz, Álvaro Del Carmen Aguayo-Muela, Krizia Radesca, Guillermo Muñoz-Andradas, Diego Domínguez-Balmaseda

**Affiliations:** 1 Faculty of Sport Sciences, Universidad Europea de Madrid, 28670 Madrid, Spain; fervasq22@gmail.com (F.V.-D.); alvaroaguayo2016@gmail.com (Á.D.C.A.-M.); krizia.radesca@universidadeuropea.es (K.R.); guillermo.munoz@universidadeuropea.es (G.M.-A.); 2 Real Madrid Graduate School, Faculty of Sports Sciences, Universidad Europea de Madrid, 18071 Madrid, Spain; 3 Faculty of Sport Sciences, Universidad de Granada, 18071 Granada, Spain

**Keywords:** eating disorder, football, EAT-26, academy, Liga MX

## Abstract

**Objectives:** Examine the prevalence of risk behaviors for the development of eating disorders in academy footballers of the Liga MX, compare sociodemographic data and highlight the participants’ perceptions regarding the influence of the sports environment and external pressure on their relationship with food and their bodies. **Methods:** A cross-sectional study was carried out with 536 footballers (331 men and 205 women) in the categories U14, U16, U18 men and U15, U19 women of Liga MX with prior consent from their clubs and strict confidentiality measures. The data were collected via Google Forms using the Eating Attitudes Test (EAT-26). **Results:** Of the participants, 13.4% met or exceeded the cut-off point on the EAT-26. The highest prevalence was observed in the Female U19 category. The reflections indicated that the sports environment and external pressure affect the relationship with food and bodies in a significant proportion of participants. **Conclusions:** The study highlights a high prevalence of risk behaviors for eating disorders in Liga MX football players, with a higher incidence in the female categories, also evidencing a multifactorial etiology.

## 1. Introduction

Eating Disorders (EDs) are a group of mental illnesses that are distinguished by the presence of altered behaviors with respect to eating habits and excessive concern for their weight or body image [1]. While EDs have been extensively studied in sports where maintaining a low body weight is critical, such as gymnastics or ballet [2,3], it is important to consider that other sports, including football, also present risk factors due to the high physical demands and pressures athletes face. Its etiology is multifactorial, taking into account the combination of genetic, environmental and personal factors, where the specific psychological factors of the sports discipline are involved [3,4]. Diagnosis is made according to the criteria defined in the Diagnostic and Statistical Manual of Mental Disorders (DSM-V), which establishes the different types of ED [1].

Previous research has shown both similarities and differences in the prevalence of EDs between athletes and non-athletes. Athletes may face higher risks due to the performance-related pressures of their sport, particularly in competitive environments. However, non-athletes, especially adolescents, are also vulnerable due to societal and media pressures, which similarly impact body image and eating behaviors. Studies comparing athlete and non-athlete groups have shown that both populations can exhibit significant risks for EDs, though the underlying factors may differ [5].

The importance of body composition in sports performance is well recognized, particularly in football, where physical demands such as endurance, strength and speed are critical [6]. This study aims to explore how these demands, combined with external pressures, impact the relationship between body composition and the development of ED behaviors in football players, a population that has received less attention in ED research. The choice to focus on footballers in this study is based on the growing recognition that body image concerns and ED behaviors are not limited to sports with explicit weight control but may also affect athletes in endurance-based team sports like football. By examining this underexplored group, this study seeks to fill a gap in existing literature and provide valuable insights into the prevalence of ED risk behaviors in youth footballers.

The sports field has several risk factors that contribute to the appearance of EDs [7]. First, there are risk factors for behaviors related to personality traits frequently found in athletes, such as perfectionism and competitiveness [8], which can be expressed as excessive attention to their diet and training or to the use of substances and/or strategies to improve their image and performance. There are also external factors that place an extra burden on the athlete [9,10] such as pressure from coaches, parents, teammates or that are caused by the sport itself, such as competition, overtraining, injuries, early initiation of specific sports training [11] or the level of competition, since athletes competing at higher levels show more signs of body dissatisfaction and ED symptoms than those at lower levels [12].

It has been suggested that athletes who compete in disciplines where low weight is important have a higher risk of presenting an ED than athletes from other disciplines [2,13]. For this reason, sports that do not fall into this classification have received less interest in the study of ED prevalence. However, football players, particularly in youth categories, face significant physical demands and external pressures related to body image and performance, even if low body weight is not the primary focus [11]. These pressures make it critical to study ED prevalence in football, a sport that has received less attention in ED research compared to weight-sensitive sports.

The International Olympic Committee has repeatedly emphasized the importance of protecting the physical and psychological safety of all athletes, including elite adolescents [14]. In line with the IOC’s recommendations, the implementation of multidisciplinary teams in sports clubs to monitor, educate and identify ED risks early is essential for safeguarding athletes’ health and performance [15]. This study contributes to this effort by examining the prevalence of risk behaviors for the development of EDs in youth football players of Liga MX, providing critical insights into an underexplored population.

## 2. Materials and Methods

### 2.1. Study Design

In the present study, a cross-sectional study was conducted between January and March of 2024, where information was collected to be analyzed qualitatively and quantitatively. The aim of this design was to assess the prevalence of risk behaviors related to EDs among youth football players and to explore the potential associations between these behaviors and sociodemographic factors, such as age, gender and competition level.

### 2.2. Participants

The participants were selected from among the players of the Liga MX youth teams. Out of a total of 18 registered clubs, 14 agreed to be part of the study. These were recruited through an initial contact with the nutrition and sports science department corresponding to each club.

#### 2.2.1. Inclusion Criteria

Players had to be registered in the Clausura 2024 Tournament in the U-14, U-16 and U-18 categories of Liga MX and in the U-19 category of Liga MX Femenil for women. The U-15 women’s category was included despite the absence of an official tournament for this group, meaning these players did not have an official registration.

#### 2.2.2. Exclusion Criteria

A total of 33 responses were excluded from analysis where age was a determining factor due to inconsistencies in the registration of the date of birth. These responses were excluded only from the age-related analysis to ensure data accuracy. The remaining sample of 503 participants was sufficient to conduct robust statistical analyses, ensuring that the exclusions did not significantly impact the study’s overall results.

A final sample of 536 participants (331 men and 205 women) was included in the study. For calculations that considered age, the sample size was 503 participants (317 men and 186 women), after excluding responses with inconsistencies.

### 2.3. Sample Size Calculation

The sample size was calculated with the statistical software G*power 3.1 considering one-tail comparisons for the exact test of binomial proportion, with an Alpha value of 0.05, a statistical power of 0.95 and an effect size of 0.1, in which the estimated minimum relevant sample was 268 participants, which was exceeded with the final sample of 536 participants.

### 2.4. Instruments

In order to detect risk behaviors for the development of ED, the Eating Attitudes Test (EAT) was administered in its short version composed of 26 items; the EAT-26 was developed and introduced by Garner, et al. (1982) [16] who were the same creators of the complete version of the EAT, which consists of 40 items. The EAT-26 is highly correlated with EAT-40 (r = 0.98) and offers a significant advantage related to the lower number of items, making it more practical for large-scale studies. Although originally designed as a diagnostic tool for anorexia, it quickly gained widespread use in both research and clinical evaluation of individuals with various degrees and types of eating disorders [17].

Its validity and reliability have been widely demonstrated in various populations, including adolescents and athletes [16,18], which makes it suitable for the present study. Its concise format and straightforward administration made it particularly useful for our study population of youth football players, as it allows for the early detection of risk behaviors in a time-efficient manner [19]. Given the competitive nature of football and the potential for disordered eating behaviors in athletes, the EAT-26 was the most appropriate instrument for this population.

In addition, two optional open-ended reflection questions designed to allow them to further explore their own perceptions and beliefs towards food and their bodies were included: (1) Do you think that the sports environment in which you find yourself and external pressure affect your relationship with food? Why? (2) Do you think that the sports environment you are in, and the external pressure affect your relationship with your body? Why? These questions provided a qualitative perspective complementary to the quantitative data collected through the EAT-26.

### 2.5. Data Collection

This study was conducted with the prior authorization and consent of the clubs and those in charge of each category: nutritionist, head of sports science and/or sports director. Strict measures were put in place to protect the confidentiality of participants and their clubs. Each player created a unique code consisting of the last three digits of their cell phone number, followed by the initial of their father’s second surname and the initial of their mother’s second surname. These codes were used in place of their real names at all stages of the study, thus ensuring their complete anonymity.

Data collection was facilitated through Google Forms (Google LLC, Mountain View, CA, USA). A link was provided to each contact person at the participating clubs, who then distributed it to the eligible players.

### 2.6. Data Analysis

Various statistical methods were employed to analyze the data collected. All statistical analyses were conducted using IBM SPSS Statistics version 25 and Microsoft Excel Version 16.89.1, while G*Power 3.1 was used to calculate the required sample size.

First, Gaussian bell curves were generated to evaluate the normal distribution of the scores obtained in each category, determining if the test scores followed a normal distribution in each subgroup of interest. To examine the relationships between the variables, Pearson correlations were calculated between the EAT-26 scores and the age of the participants. Correlation analyses were conducted separately for male and female categories, as well as for the total sample, to explore the association between age and the presence of risk behaviors for the development of ED. Additionally, a Multivariate Analysis of Variance (MANOVA) was performed to investigate differences in EAT-26 scores, considering gender, age and football category. This allowed for the simultaneous examination of multiple dependent variables related to eating disorder risk behaviors. The significance level was set at *p* < 0.05 for all analyses.

## 3. Results

**Figure 1:**
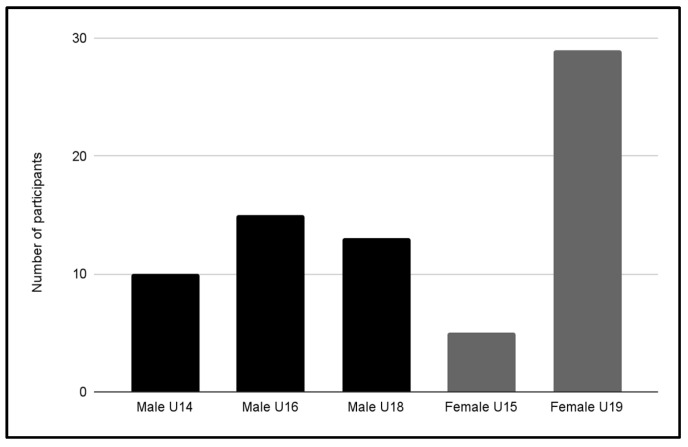
Participants with a score ≥ 20.

The average age of the participants was 16.5 ± 1.2 years, with ages ranging from 14 to 19 years. Of the total sample, 13.4% met or exceeded the cut-off point on the EAT-26. The highest prevalence was observed in the Female U19 category (22.6%). Figure 1 shows the distribution by category of participants with this score.

**Figure 2:**
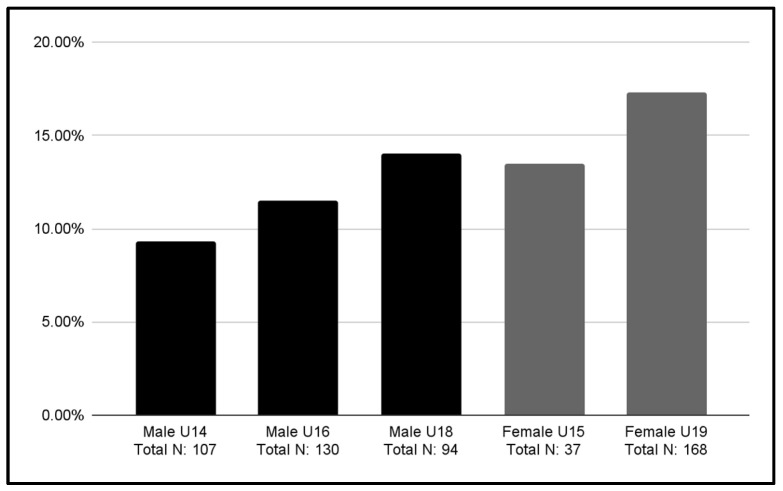
Percentage of participants with a score ≥ 20 relative to their own category.

For a more precise understanding of these cases in relation to the total number of responses recorded in each category, Figure 2 presents the data in the form of a percentage.

### 3.1. Normal Distributions of EAT-26 Test Scores

#### 3.1.1. Normal Distributions by Category

**Figure 3:**
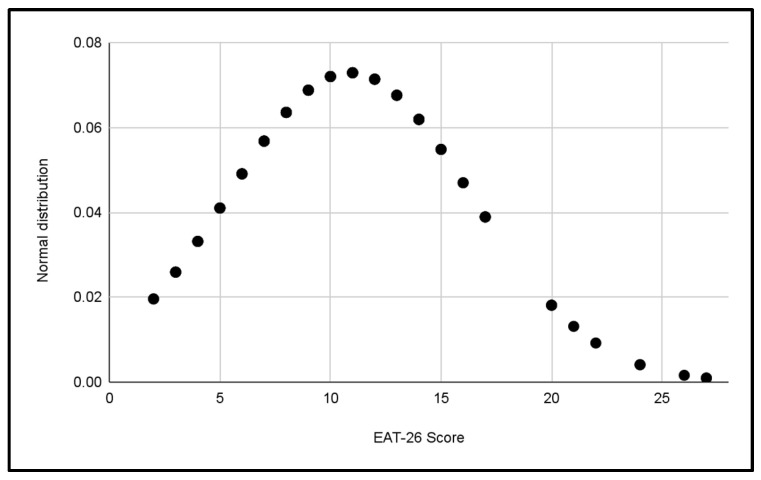
Normal distribution of EAT-26 scores for the U14 Male category.

**Figure 4:**
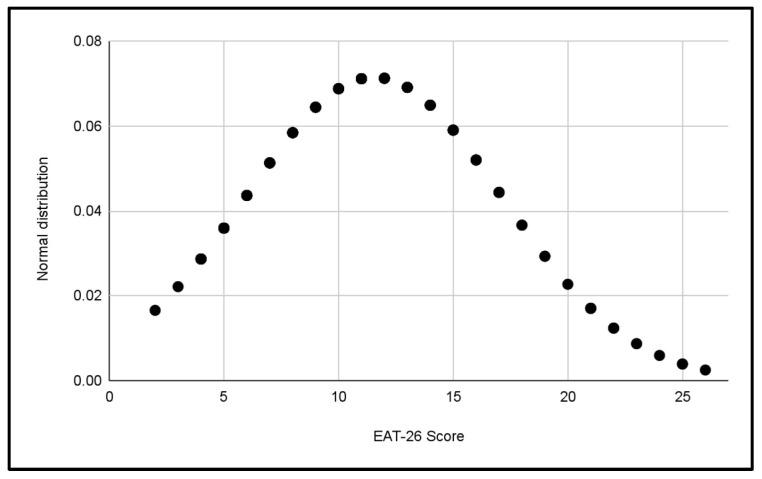
Normal distribution of EAT-26 scores for the U16 Male category.

**Figure 5:**
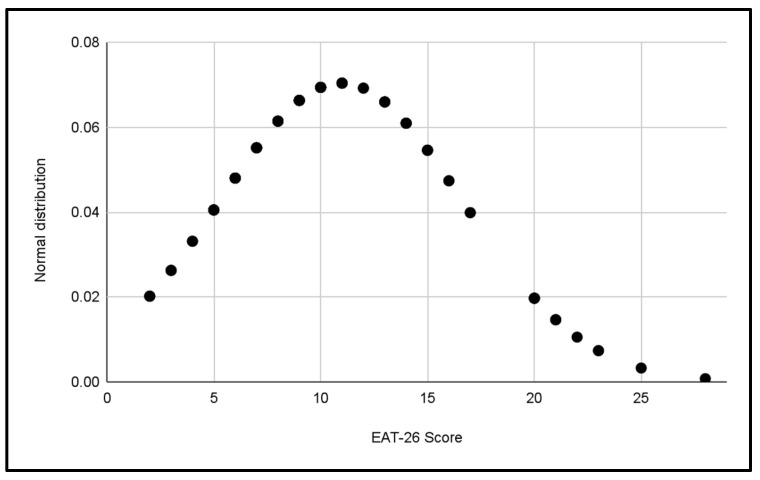
Normal distribution of EAT-26 scores for the U18 Male category.

**Figure 6:**
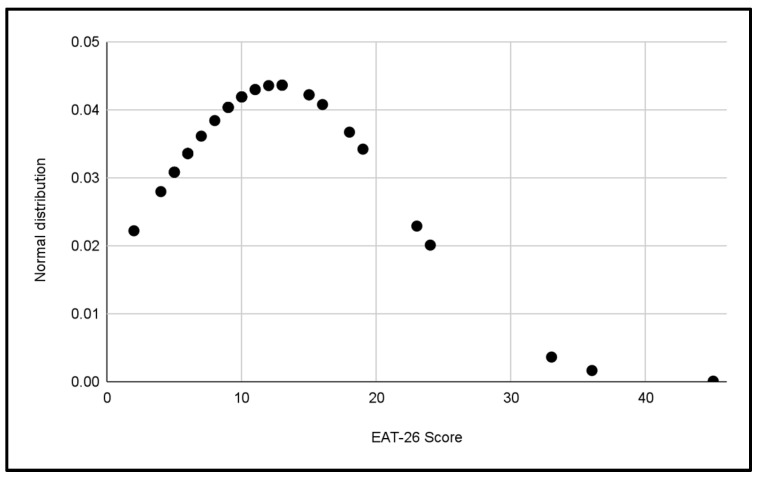
Normal distribution of EAT-26 scores for the U15 Female category.

**Figure 7:**
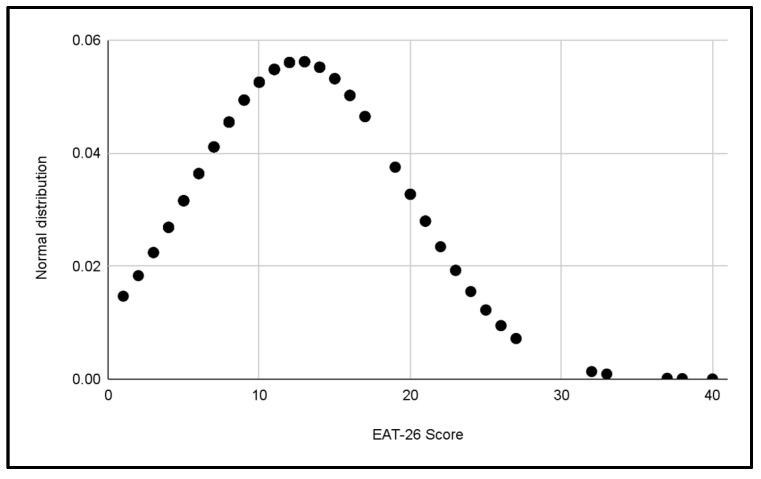
Normal distribution of EAT-26 scores for the U19 Female category.

Five normal distributions were analyzed to evaluate the scores of the EAT-26 Test in the different categories of the study, which can be seen in Figure 3, Figure 4, Figure 5, Figure 6 and Figure 7.

In each distribution, notable differences in scores were observed, these figures indicate the presence of cases with a significant risk of problematic eating behaviors, especially in the female categories, where the highest scores were recorded.

Differences in the variability of scores within each category were also observed.

#### 3.1.2. Normal Gender Distributions

**Figure 8:**
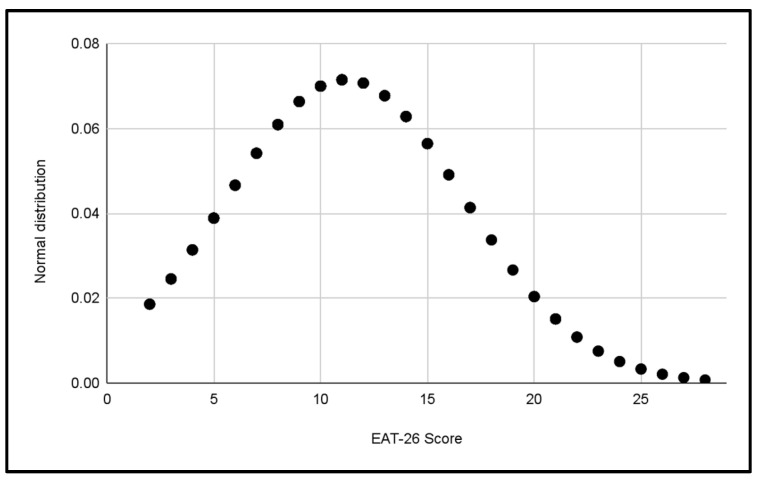
Normal distribution of EAT-26 scores for Male categories.

**Figure 9:**
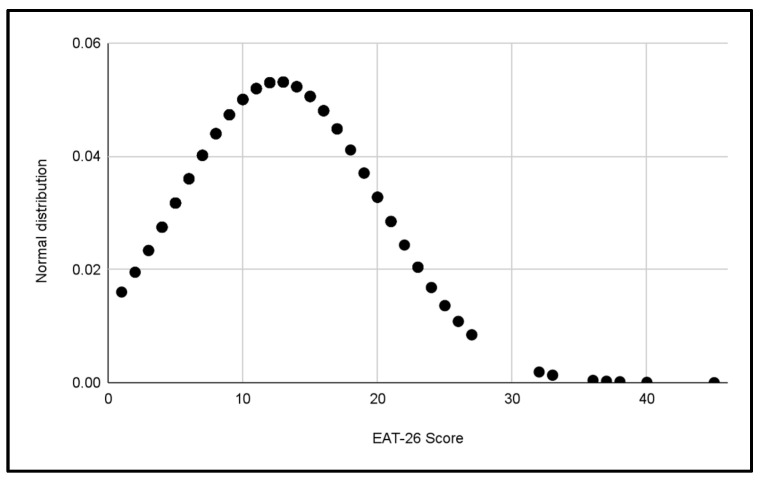
Normal distribution of EAT-26 scores for Female categories.

Two additional normal distributions were performed to compare the variability of EAT-26 scores between the male and female categories in Figure 8 and Figure 9. These data suggest a greater dispersion of scores in the women’s categories, which could reflect a wider variety of eating behaviors among female footballers compared to male footballers.

#### 3.1.3. Normal Distribution of the Total Sample

**Figure 10:**
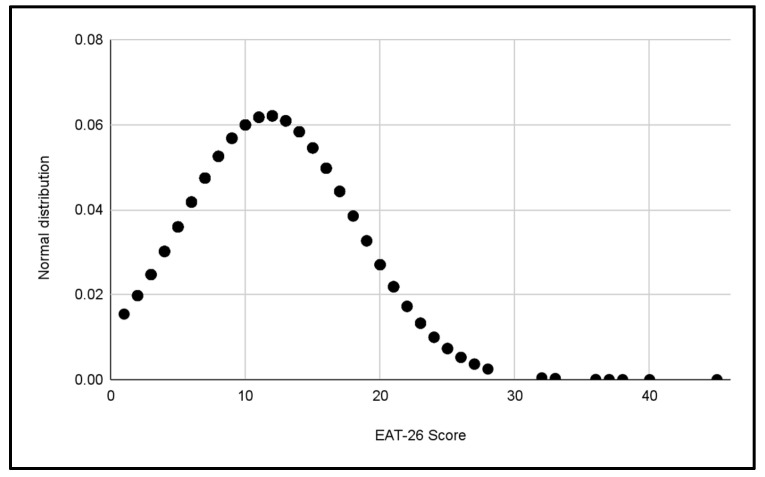
Normal distribution of EAT-26 scores for the total sample.

In the same way, a normal distribution was carried out that evaluated the EAT-26 scores of the total sample, which can be seen in Figure 10.

When looking at the distribution, scores that oscillated around the mean (11.71) stand out, suggesting a moderate prevalence of risky eating behaviors in the sample. However, it is important to note that, although the mean does not reflect a significant score, there is still a considerable number of participants who obtained that score, represented by the values on the far right of the distribution bell.

### 3.2. Correlations

#### 3.2.1. Pearson Correlations Women’s Categories

The correlation analysis between the score and the age of the players revealed an R^2^ coefficient of 0.00582. Although this value is relatively low, it indicates a trend in the relationship between age and test score.

**Figure 11:**
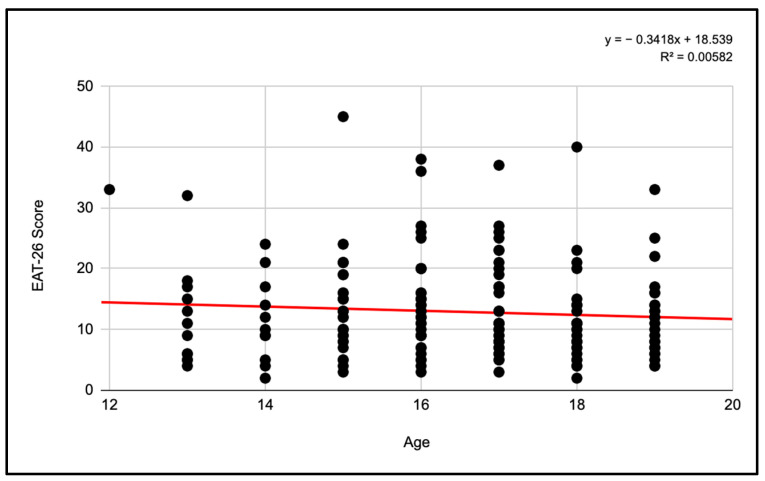
Pearson correlation of EAT-26 scores and age for female participants.

The resulting regression equation shows that, on average, the score decreases by 0.3418 points for each additional year of age. However, it is important to note that age explains only a fraction of the variability observed in the test scores, suggesting the presence of other influential factors (Figure 11).

#### 3.2.2. Pearson Correlations Men’s Categories

In contrast to the results obtained in the women’s categories, the correlation between the score and the age of the players revealed an R^2^ coefficient of 0.00028, resulting in an extremely low value.

**Figure 12:**
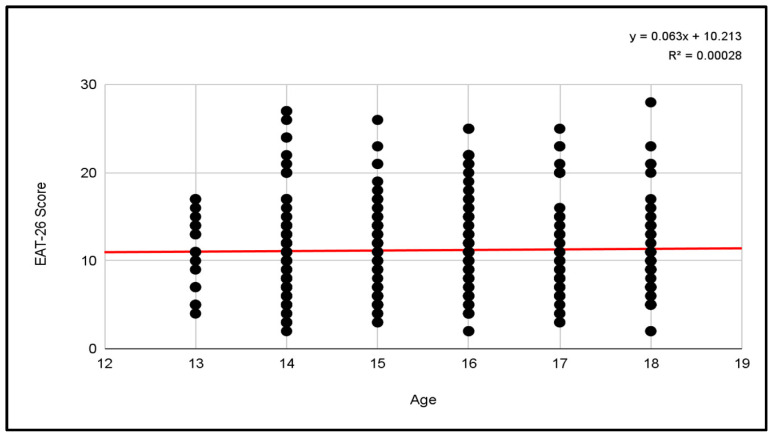
Pearson correlation of EAT-26 scores and age for male participants.

The regression equation suggests that, on average, the score on the EAT-26 increases by 0.063 points for each additional year of age. However, this relationship is almost non-existent, suggesting that age has a minimal influence on test scores in this population and that other factors may be exerting a greater influence (Figure 12).

#### 3.2.3. Pearson Correlations Total Sample

When examining the correlation between score and age in the total sample, an R^2^ coefficient of 8.8 × 10^−5^ was found, thus being an extremely low value.

**Figure 13:**
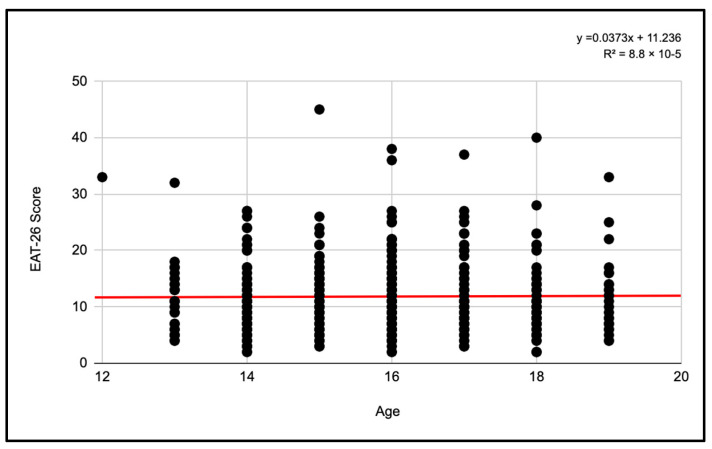
Pearson correlation of EAT-26 scores and age for the total sample.

The regression equation suggests that, on average, the score on the EAT-26 test increases by 0.0373 points for each additional year of age. However, the negligible relationship between age and test scores suggests that other factors must be exerting a much greater influence on the results obtained (Figure 13).

### 3.3. Multivariate Analysis of Variance

Multivariate analysis of variance (MANOVA) was used in Table 1 to examine the relationship between sociodemographic data and the EAT-26 score.

A significant effect of age was found on the test score, taking into account the level of significance established in this study, suggesting that age is significantly associated with eating disorder risk behaviors. On the other hand, no significant effects of category or gender were found on the test score.

These findings suggest that the relationship between sociodemographic data and risk behaviors is more complex, and it is possible that other factors are influencing the manifestation of risk behaviors.

### 3.4. Reflective Analysis

In addition to the quantitative results presented above, the questionnaire included two optional open-ended questions, for the first question: “Do you think that the sports environment in which you find yourself and external pressure affect your relationship with food? Why?” 514 responses were registered, of which 38.1% said yes. While, in the second question: “Do you think that the sports environment in which you are, and the external pressure affect your relationship with your body? Why?” 491 responses were registered, of which 37.4% said yes.

A comprehensive analysis was conducted focusing exclusively on the responses of participants who had answered “yes” to the questions mentioned above. This approach was designed to further explore the perceptions and experiences of those participants who recognized the influence of the sports environment and external pressure on their relationship with food and their bodies.

**Figure 14:**
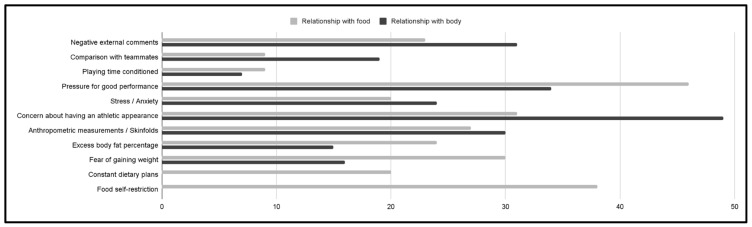
Reflections: Factors of the Sports Environment and External Pressure Affecting Footballers’ Relationship with Food and Their Bodies.

Keywords that were repeated in different responses were identified, as well as those that referred to the same factors. These terms were recorded and the number of times they were mentioned was meticulously documented. This information was then used to exemplify the frequency of each factor in Figure 14.

## 4. Discussion

The analysis of the sample revealed that 13.4% of the subjects evaluated reached or exceeded the cut-off point (≥20 points) in the EAT-26, with a prevalence of 11.4% in men and 16.5% in women, highlighting a significant number of risky eating behaviors in youth football players. These results are particularly important, as they indicate that disordered eating behaviors are present even in a sport like football, which is not traditionally classified as weight-sensitive.

Comparing these findings to previous studies, we observe that the prevalence in our study is higher than that found in other sports disciplines. For example, Schaal et al. (2011) [20] reported a prevalence of 4% in men and 6.5% in women across multiple sports, specifically mentioning that the group of women with the lowest prevalence were those who competed in team ball sports, with 5.8%, while Martinsen et al. (2013) [21] found a prevalence of 3.2% in men and 10.8% in women in elite athletes from various disciplines. The higher prevalence observed in football players in this study suggests that the pressures athletes face, even in non-weight-sensitive sports, can significantly impact their eating behaviors. Gender differences were also relevant, with women showing a higher prevalence of risky eating behaviors than men. This aligns with findings from other studies that show women are generally at higher risk for developing eating disorders, even in team-based, endurance sports.

Another significant finding of the study is the small relationship between age and ED risk behaviors. While the overall effect of age on eating behaviors may appear minimal, it suggests that younger athletes may be more vulnerable to certain pressures related to body image and food. These findings are consistent with studies by Baldó et al. (2019) [22] and Petisco et al. (2020) [23], which highlight that the risk of disordered eating can be present across all ages in athletes, though its manifestation may vary depending on age and other factors.

The influence of the sports environment and external pressures on athletes’ eating behaviors was another key finding of this study. Participants reflected on the impact of body image pressures, competition and external expectations on their relationship with food and their bodies. These reflections are similar to the conclusions of Stirling et al. (2012) [24] who highlighted the importance of both external and internal pressures in shaping athletes’ eating behaviors.

In addition, Conlin et al. (2021) [25], Holland et al. (2016) [26] and Fatt et al. (2024) [27] found that social media plays a crucial role in promoting unrealistic body ideals, particularly among younger athletes who are more susceptible to body image concerns. Exposure to such content can increase the risk of disordered eating behaviors, even in sports that do not traditionally emphasize weight control. Addressing the influence of social media is essential, particularly for youth athletes who may be vulnerable to these pressures.

These findings demonstrate the need for structured educational and nutritional programs to address disordered eating behaviors in youth football players, as well as the importance of early intervention and multidisciplinary approaches.

This study has several strengths; it focuses on a population that has been underexplored in previous research, providing valuable insights into disordered eating behaviors in youth football players which is an area that has traditionally received less attention compared to athletes in weight-sensitive sports. The large sample size, as well as the inclusion of both male and female participants, enhances the generalizability of the findings and allows for meaningful comparisons across genders.

However, there are limitations that should also be acknowledged. First, although self-administered questionnaires are a very useful tool for assessing eating behaviors, clinical interviews are considered more accurate in diagnosing eating disorders. In addition, the standard questionnaires used may not be sensitive enough to detect specific symptoms in athletes, as they are designed for the general population. In the same way, it is essential to standardize the methodology of studies on ED symptoms to obtain comparable results. To do this, it will be necessary to develop validated questionnaires specifically for the athlete population.

As for future lines of research, it would be valuable to carry out long-term follow-up studies to determine if the results obtained are maintained or change over time. It would also be very interesting to further explore the impact of other factors, such as anxiety, perfectionism, motivation or self-esteem using clinically validated questionnaires, as well as body composition, playing position and lifestyle variables, in order to provide a more comprehensive understanding of the multifactorial nature of EDs in athletes, thus contributing to the establishment of more effective prevention and treatment strategies in this population.

## 5. Practical Implications and Nutritional Interventions

Given the findings of this study, it is crucial to implement structured nutritional interventions that not only address disordered eating behaviors but also promote overall health and performance in football players.

Football is a high intensity, endurance-based sport that requires optimal nutrition to support physical performance, recovery and injury prevention, which is why individualized nutritional plans are essential. These plans should be based on the player’s position, training load and specific goals. Ensuring adequate intake of macronutrients is critical for sustaining the high energy demands and supporting muscle recovery. However, it is also important that these plans include a degree of flexibility to avoid the negative psychological impact of overly rigid diets, which may lead to unhealthy relationships with food [25].

Nutritional interventions should be integrated into a multidisciplinary approach involving collaboration between nutritionists, coaches, doctors and sport psychologists. By working together, these professionals can ensure that players receive comprehensive support in managing both physical and mental demands [28]. A structured education program focused on healthy eating habits and the risks of disordered eating is essential to prevent the onset of ED behaviors [29].

Effective implementation of these interventions in sports clubs requires the commitment of club management and coaching staff. Regular monitoring of players’ nutritional habits and body composition, combined with ongoing education, can help create a supportive environment where healthy eating is prioritized. Team-based nutritional workshops and one-on-one consultations can be used to deliver personalized guidance, making it easier for players to adhere to nutritional recommendations [30].

## 6. Conclusions

This study reveals a worrying prevalence of risk behaviors for the development of eating disorders among Liga MX youth players, with a prominent incidence in the women’s categories. In addition, the complexity of sociodemographic factors and the perception of athletes on the influence of the sports environment and external pressure on their relationship with food and their bodies were evidenced. These findings underscore the need to implement specific prevention and treatment measures targeting this sports population. However, the importance of future research to deepen this topic is recognized.

## Figures and Tables

**Table 1:** Multivariate Analysis of Variance of the Relationship Between Sociodemographic Data and EAT-26 Scores.

Origin	Sum of Squares Type III	df	Mean Squares	F	Sig
**Corrected model**	1550.251 ^a^	25	62.010	1.498	0.059
**Intercept**	8376.176	1	8376.176	202.309	<0.001
**Age**	692.849	7	98.978	2.391	0.021
**Category**	288.399	4	72.100	1.741	0.140
**Gender**	3.258	1	3.258	0.079	0.779
**Error**	19,749.129	477	41.403		
**Total**	91,139.000	503			
**Corrected total**	21,299.380	502			

^a^ R squared = 0.073 (Adjusted R Squared = 0.024).

## Data Availability

Data are contained within the article.
